# Scavenger properties of cultivated pig liver endothelial cells

**DOI:** 10.1186/1476-5926-3-4

**Published:** 2004-08-12

**Authors:** Kjetil H Elvevold, Geir I Nedredal, Arthur Revhaug, Bård Smedsrød

**Affiliations:** 1 Department of Experimental Pathology, Institute of Medical Biology, University of Tromsø, 9038 Tromsø, Norway; 2 Department of Digestive Surgery, University Hospital of Tromsø, 9038 Tromsø, Norway

## Abstract

**Background:**

The liver sinusoidal endothelial cells (LSEC) and Kupffer cells constitute the most powerful scavenger system in the body. Various waste macromolecules, continuously released from tissues in large quantities as a consequence of normal catabolic processes are cleared by the LSEC. In spite of the fact that pig livers are used in a wide range of experimental settings, the scavenger properties of pig LSEC has not been investigated until now. Therefore, we studied the endocytosis and intracellular transport of ligands for the five categories of endocytic receptors in LSEC.

**Results:**

Endocytosis of five ^125^I-labelled molecules: collagen α-chains, FITC-biotin-hyaluronan, mannan, formaldehyde-treated serum albumin (FSA), and aggregated gamma globulin (AGG) was substantial in cultured LSEC. The endocytosis was mediated via the collagen-, hyaluronan-, mannose-, scavenger-, or IgG Fc-receptors, respectively, as judged by the ability of unlabelled ligands to compete with labelled ligands for uptake. Intracellular transport was studied employing a morphological pulse-chase technique. Ninety minutes following administration of red TRITC-FSA via the jugular vein of pigs to tag LSEC lysosomes, cultures of the cells were established, and pulsed with green FITC-labelled collagen, -mannan, and -FSA. By 10 min, the FITC-ligands was located in small vesicles scattered throughout the cytoplasm, with no co-localization with the red lysosomes. By 2 h, the FITC-ligands co-localized with red lysosomes. When LSEC were pulsed with FITC-AGG and TRITC-FSA together, co-localization of the two ligands was observed following a 10 min chase. By 2 h, only partial co-localization was observed; TRITC-FSA was transported to lysosomes, whereas FITC-AGG only slowly left the endosomes. Enzyme assays showed that LSEC and Kupffer cells contained equal specific activities of hexosaminidase, aryl sulphates, acid phosphatase and acid lipase, whereas the specific activities of α-mannosidase, and glucuronidase were higher in LSEC. All enzymes measured showed considerably higher specific activities in LSEC compared to parenchymal cells.

**Conclusion:**

Pig LSEC express the five following categories of high capacity endocytic receptors: scavenger-, mannose-, hyaluronan-, collagen-, and IgG Fc-receptors. In the liver, soluble ligands for these five receptors are endocytosed exclusively by LSEC. Furthermore, LSEC contains high specific activity of lysosomal enzymes needed for degradation of endocytosed material. Our observations suggest that pig LSEC have the same clearance activity as earlier described in rat LSEC.

## Background

Livers of pig are used in a wide range of experimental settings related to problems encountered in human medicine such as fulminant hepatic failure [1,2] and graft survival in liver transplantation [3]. Bioartificial liver support systems, which combine living cells of the liver in an extracorporeal circuit, have been successfully used in first clinical trials [4]. The shortage of human organs to be used for bioreactors and the lack of safe and effective human liver cell lines have resulted in pigs becoming an important hepatic cell source. Although much work based on the use of pig liver has been carried out in the past, no studies have so far focused on isolated cell populations of the liver. To overcome this lack of knowledge, we recently published a study on high yield isolation and characterization of pig liver cells [5]. This work represents a basis for *in vitro *studies of the cells constituting the hepatic reticulo-endothelial system: the Kupffer cells (KC) and the liver sinusoidal endothelial cells (LSEC) [6]. Together these cells represent the most powerful scavenger system of the body. Most soluble or insoluble macromolecular harmful material entering the body from the gut is transported to the liver via the portal vein and eliminated by uptake in KC and LSEC. Studies in rats have revealed that LSEC are actively engaged in blood clearance of an array of waste macromolecules of both soluble and colloidal nature. Major components of connective tissue as well as other potentially hazardous products are eliminated rapidly and almost exclusively from the blood by receptor-mediated endocytosis in LSEC [7,8].

The endocytic receptors of rat LSEC can be listed in five categories: (i) collagen α-chain receptor (COLLAR), (ii) hyaluronan receptor (HAR), (iii) IgG Fc receptor, (iv) mannose receptor, and (v) scavenger receptor (SR). With specificity for free α-chains of types I, II, III, IV, V, XI collagens, but not native triple helical collagen, the COLLAR function has so far only been described in LSEC [9,10]. The HAR is responsible for the clearance of circulating hyaluran (HA) [11,12]. Chondroitin sulphate, dermatan sulphate, as well as some ligands for the SR are also endocytosed via this receptor, which has been purified and characterized [13,14]. In both KC and LSEC the IgG Fc receptor has been shown to mediate the elimination of circulating IgG immune complexes in the liver [15-18]. The mannose receptor recognizes the terminal non-reducing sugar residue of the oligosaccharide (fucose, mannose or N-acetyl-glucosamine) moiety of glycoproteins [19,20]. First found in alveolar macrophages [21], this receptor was later functionally demonstrated on LSEC as well [22], where it performs an extremely rapid endocytosis of mannosylated glycoproteins [23]. Sharing the feature of recognizing negatively charged ligands, the SR is a rapidly growing family of receptors that have been identified in macrophages and other cell types [24,25]. An array of physiological and foreign ligands has been reported to be eliminated from the circulation predominantly via endocytosis in the rat LSEC SR [8,26-29], using the receptors described above. Recent observations have resulted in new insight into the LSEC SRs and a relationship between SR and the HAR. First, it was published that a rat LSEC surface protein has affinity for both HA and SR-ligands; Antibodies generated against this protein inhibited endocytosis of both HA and various SR-ligands [13]. Therefore, the name hyaluronan/scavenger receptor (HA/S-R) was coined to describe its activity [14,30], but it is also known as stabilin-2. It was recently reported that LSEC in knock-out mice lacking SR class A, remove SR-ligands both *in vivo *and *in vitro *as avidly as do wild-type mice [31]. These results show that LSEC, employing this unique receptor, represents the most important cellular site of uptake of blood-borne SR-ligands.

We have previously shown that pig LSEC express a functionally active receptor that both *in vivo *and *in vitro *clear a SR ligand [5]. Until now this represents the only functional study that has been done on pig LSEC. Accordingly, in the present study we wanted to study if pig LSEC carry out the same avid clearance based on the use of the same endocytosis mechanisms that has been already shown for the rat. We also compared the technique of isolation of pig LSEC with the established method used to prepare isolated rat LSEC [32]. Apart from the difference in organ size, the most striking difference from rat LSEC isolation was the high number of stellate cells within the pig non-parenchymal cells (NPC) which hampered the purification of the pig LSEC. In addition to represent a functional study of pig LSEC, this study also describes a method for obtaining a high number of purified pig LSEC which is necessary for the further purification and characterisation of molecules unique for LSEC.

We here show that pig LSEC, but not stellate cells, parenchymal cells (PC) or KC, express the same five categories of endocytosis receptors for soluble and colloidal ligands as reported for rat LSEC. Ligands endocytosed via these receptors are transported along the endocytic pathway and degraded. Assays carried out to measure activity of lysosomal enzymes in cells showed that several enzymes are present at higher specific activities in LSEC than in either KC or PC.

## Results

### Isolation and cultivation of liver cells

The fraction of cells collected after density centrifugation in OptiPrep consisted of LSEC, stellate cells, KC, PC and a minor proportion of unidentified cells, possibly of epithelial nature. In this fraction, LSEC were most numerous, closely followed by stellate cells. Further purification, using either elutriation centrifugation or selective adherence, resulted in 80–95 % purity of LSEC. One cycle of elutriation centrifugation resulted in approximately 10^8 ^LSEC. Contaminants in LSEC cultures were epithelial-like cells, stellate cells, KC, and PC. Compared with rat and mice PC, the population of PC from pig are much more heterogeneous in size; consequently, pig LSEC cultures may be contaminated by small PC.

Cultures of KC prepared by selective adherence were prepared by incubating the non-PC fraction, for 15 min, on glass dishes coated with glutaraldehyde-treated BSA This procedure yielded 70–80 % pure KC. The use of tissue culture plastic dishes resulted in a lower purity of KC (40–50%), with a higher proportion of LSEC.

### Specificity of endocytosis via different receptors in cultured LSEC

**Figure 1:**
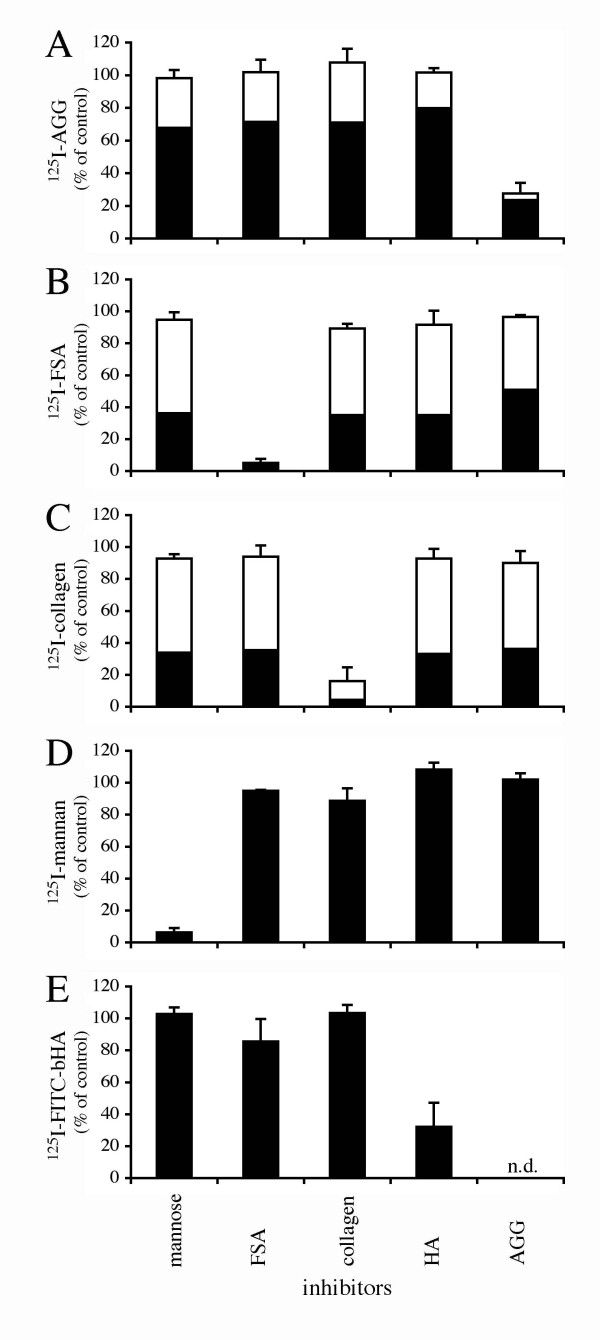
**Receptor specificity. **Specificity of uptake of ^125^I-AGG (A), ^125^I-FSA (B), ^125^I-collagen (C), ^125^I-mannan (D), or ^125^I-FITC-bHA (E) in LSEC in the presence of mannose (50 mM), FSA (0.1 mg/ml), COLLA (0.1 mg/ml), hyaluronan (0.1 mg/ml) and AGG (0.3 mg/ml). Uptake (cell-associated radioactivity (solid bars) plus acid soluble radioactivity in spent medium [open bars]) was measured after 120 min incubation at 37°C. Results, given as % of control, are means of three experiments, each consisting of three parallels. Error bars represent standard deviation (+SD).

Experiments were carried out to establish whether ligands known to be taken up by rat LSEC via the different categories of receptors are endocytosed with the same specificities in pig LSEC. To this end, trace amounts of ^125^I-labelled AGG, FSA, collagen α-chain (COLLA), mannan and FITC-bHA were incubated with purified cultures of pig LSEC with or without the presence of excess amounts of unlabelled substances known to compete with the radiolabelled ligands for endocytosis in rat LSEC. Control experiments showed that endocytosis was substantial following 2 h incubation at 37°C with only radiolabelled ligands. Expressed as % endocytosis of total added ligand, the results were as follows: AGG, 7.1%; FSA, 42.7%; COLLA, 59.7%; mannan, 38.2%; FITC-bHA, 12.1% (Fig. 1). Approximately 40–50% of the endocytosed COLLA and FSA and 30% of endocytosed AGG were recovered as acid-soluble degradation products in the medium after 2 h of incubation. No acid soluble radioactive degradation products of ^125^I-mannan or ^125^I-FITC-bHA were released to the medium. Competition experiments showed that AGG (300 μg/ml) inhibited uptake of trace amounts of ^125^I-AGG by 73% (Fig. 1A). AGG at a concentration of 100 μg/ml was only 40% inhibitory (results not shown). FSA (100 μg/ml) inhibited uptake of trace amounts of ^125^I-FSA and ^125^I-FITC-bHA by 95% and 14%, respectively (Figs. 1B and 1E); COLLA (100 μg/ml) inhibited uptake of trace amounts of ^125^I-COLLA by 84% (Fig. 1C); mannose (50 mM) inhibited uptake of trace amounts of ^125^I-mannan by 96% (Fig. 1D); and hyaluronan (100 μg/ml) inhibited uptake of trace amounts of ^125^I-FITC-bHA by 62% (Fig. 1E).

### Endocytosis of fluorochrome-labelled ligands

To study the transport from early endosomes to later endocytic compartments, late endosomes and lysosomes were prelabelled with TRITC by administering TRITC-FSA to pigs via the jugular vein, 1.5 h prior to preparation of cultures [33]. Following incubation for 6.5 h at 37°C to allow the LSEC to adapt to the *in vitro *conditions, cultures were pulsed for 1 h at 4°C with fluorochrome-labelled ligands, and chased after an additional 10 min or 2 h incubation at 37°C. Due to weak intensity, FITC-bHA needed to be pulsed for 20 min at 37°C in cells that were not prelabelled with TRITC-FSA, and then chased for 20 min or 2 h to obtain a detectable image of the uptake.

**Figure 2:**
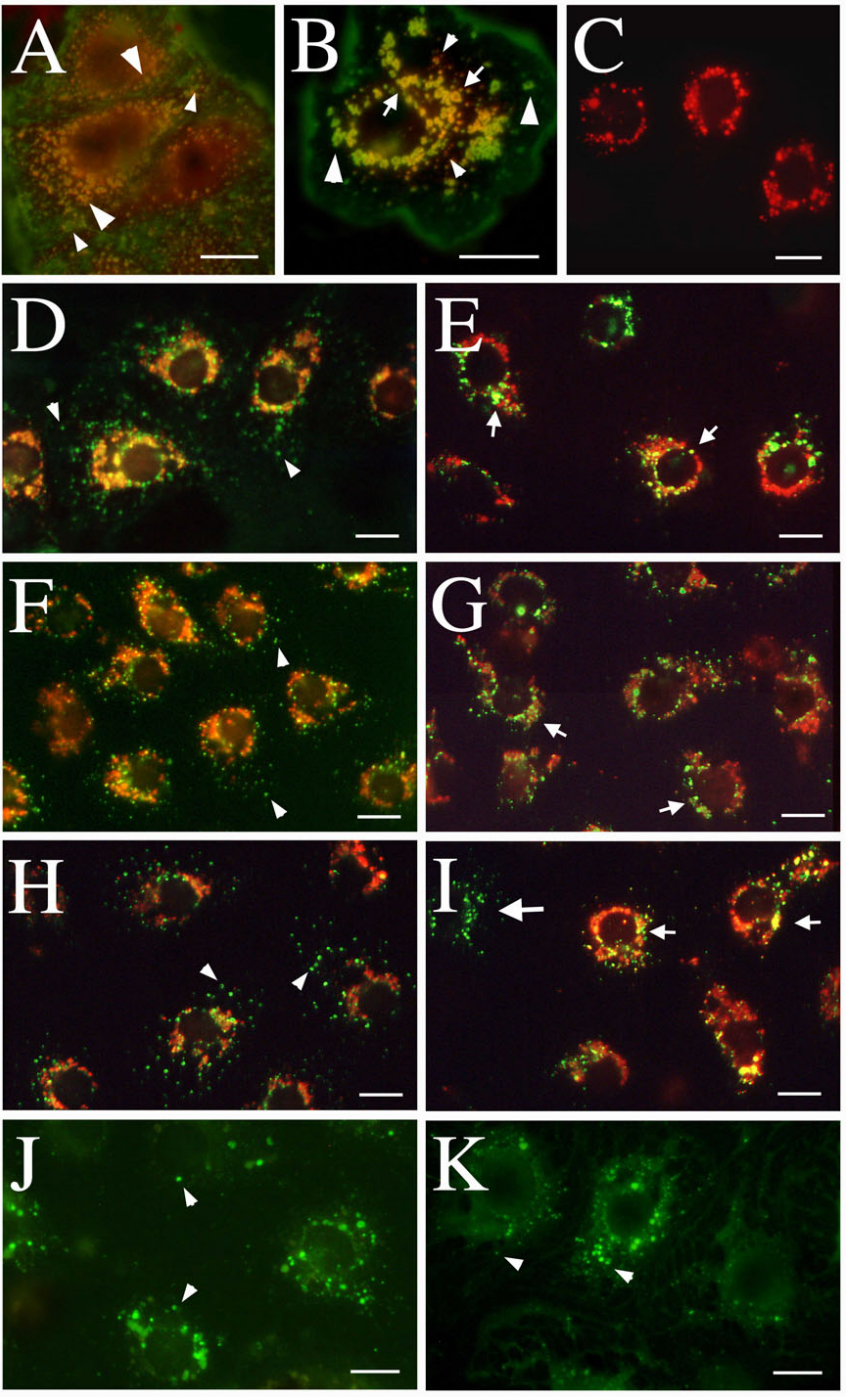
**Intracellular transport of endocytosed ligands. **Cultures of LSEC were pulsed with both TRITC-FSA and FITC-AGG for 1 h at 4°C. Chasing was performed after removal of unbound ligand by washing, and transferring of the cultures to 37°C. The cultures were fixed after chase periods of 10 min or 2 h and examined in fluorescence microscope. At 10 min (A) all TRITC-FSA co-localized with FITC-AGG (yellow colour indicates co-localization) in large ring-shaped vesicles (large arrowheads), and some vesicles with only FITC-AGG were observed (small arrowheads). After 2 h (B), co-localization of FITC-AGG and TRITC-FSA in large vesicles (arrows) was observed together with big vesicles with only FITC-AGG (large arrowheads) and small vesicles with only TRITC-FSA (small arrowheads). Controls show a more perinuclear appearance of TRITC-FSA when pulsed and chased for 2 h alone (C). In other experiments, TRITC-FSA was injected intravenously 1.5 h before isolation of the cells. Following an additional 6.5 h of cultivation at 37°C cultures of LSEC were pulsed with FITC-FSA (D-E), FITC-collagen (F-G), or FITC-mannan (H-I) for 1 h at 4°C. Following a 10 min chase, the FITC-ligands were observed to appear in small vesicles (arrowheads), and did not co-localize with TRITC-FSA (D, F and H). After 2 h, the FITC-ligands were transported to perinuclear compartments that co-localized almost completely with TRITC-FSA (small arrows in E, G and I). Other cultures of LSEC were pulsed for 10 min at 37°C with FITC-bHA. Following a 20 min chase, the FITC-bHA was observed in vesicles distributed all over the cell (arrowheads in J), and a similar appearance was observed after 2 h (arrowheads in K). Occasionally, cells that did not take up TRITC-FSA *in vivo*, but endocytosed FITC-ligands *in vitro *(big arrow in I), were observed. Scale bars: 10 μm.

Observation of these cultures in the fluorescence microscope revealed that following a 10 min chase, FITC-labelled FSA (Fig. 2D), COLLA (Fig. 2F) and mannan (Fig. 2H) were distributed in small (green) vesicles scattered throughout the cytoplasm. Co-localization of these green vesicles with perinuclear organelles prelabelled with red TRITC-FSA could not be observed at this early chase period. FITC-bHA chased for 20 min was also found in similar small vesicles and also in larger vesicles spread throughout the cell (Fig 2J). Furthermore, following 10 min chase, most FITC-labelled AGG and TRITC-FSA co-localized (yellow color indicates co-localization) in large ring shaped vesicles throughout the cell, but some vesicles with only FITC-AGG were observed (Fig. 2A). After 2 h, the co-localization with TRITC-FSA was significant for FSA (Fig. 2E), COLLA (Fig. 2G), and mannan (Fig. 2I), whereas FITC-bHA was found in vesicles similar to those observed after 20 min (Fig. 2K). After this chase-period FITC-AGG and TRITC-FSA, only partly co-localized as seen in a single cell (Fig. 2B). FITC-AGG was still present in large ring shaped vesicles, of which many still were spread throughout the cell, whereas TRITC-FSA was found in small red vesicles but also in some big ring shaped vesicles together with FITC-AGG. Interestingly, control cells chased for 2 h with only TRITC-FSA (Fig. 2C) had all ligand concentrated in the perinuclear region in lysosomes, and no ring shaped vesicles were observed. This indicates that FITC-AGG influences the transport of TRITC-FSA to the lysosomes.

The FITC-ligands were observed only in cells that endocytosed TRITC-FSA *in vivo*. In spite of the fact that nearly all cells judged as LSEC in the cultures accumulated FITC-ligands *in vitro*, approximately 5% of the cells had not taken up TRITC-FSA *in vivo*.

### Lysosomal enzyme activities

By using sensitive fluorometric assays, we measured the activities of six different lysosomal hydrolases, and compared their specific activities in three major liver cell types LSEC, KC and PC. The results, listed in Table 2, reveal that all the enzymes measured were present in significantly higher specific activities in LSEC as compared to PC, with LSEC:PC ratios as high as 7.5, 6.8, 4.9, 3.3 and 2.3 for hexosaminidase, glucuronidase, aryl sulphatase, acid lipase and acid phosphatase, respectively. The specific activities of α-mannosidase and glucuronidase in LSEC were significantly higher also when compared to KC. Compared to PC, all the enzymes except for acid lipase were present in significantly higher activities in KC.

**Table 2:** Specific activities of lysosomal enzymes in parenchymal (PC), sinusoidal endothelial cells (LSEC) and Kupffer cells (KC) isolated from pig liver.

	**LSEC**	**KC**	**PC**
α-Mannosidase (= 7)	0.76^a ^(0.14)	0.59^b ^(0,16)	0.40^c ^(0.08)
Hexosaminidase (n = 7)	30.3^a ^(7.86)	29.2^a ^(1.70)	4.02^b ^(2.06)
Acid lipase (n = 6)	6.66^a ^(2.40)	4.33^ab ^(2.60)	2.03^b ^(0.46)
Acid phosphatase (n = 6)	11.2^a ^(3.90)	8.89^ab ^(2.72)	4.97^b ^(1.96)
Glucuronidase (n = 5)	3.55^a ^(0.77)	2.35^b ^(0.53)	0.52^c ^(0.28)
Aryl sulphatase (n = 4)	0.27^a ^(0.11)	0.20^a ^(0.05)	0.05^b ^(0.02)

Each experiment consisted of three parallels. Values are means (SD). The letters (a, b, and c) indicate significant statistical differences between the values of the different cell types. Enzyme activities are expressed as 10^6 ^4-methylumbelliferyl (-mannopyranoside, -N-acetyl-glucosaminide, -oleate, -phosphate, -glucuronide, -sulfate) molecules released per min per g cell solubilisate at 37°C. (p < 0.05, ANOVA, LSD post hoc test).

## Discussion

In spite of the fact that most researchers in the field would assume that pig LSEC perform the same important scavenger function as rat LSEC [34], no studies have been published so far to actually prove this supposition. The present study was undertaken to establish whether LSEC from pig liver have the same high scavenger capacity as has been found for LSEC from rat liver [7]. To this end pig liver LSEC were isolated and cultivated, and studied with respect to endocytic and degradative activity.

Since LSEC make up just a few percent of the total liver volume [35], it is important to employ a cell marker that readily and specifically distinguishes LSEC from other types of liver cells. We found that soluble FITC- and TRITC-FSA serve this function. Although other fluorescently marked probes can be used for the same specific distinction of LSEC, FITC-FSA stands out as the optimal marker, since it is inexpensive, easy to prepare and very stable. To label LSEC *in vivo*, 20 mg FITC/TRITC-FSA in 20 ml physiological saline was administered via the left external jugular vein 90 min prior to perfusion of the liver with collagenase.

The selective adherence steps used for the separation of KC from the NPC fraction in mice (10 min on uncoated tissue culture plastic dishes) [31] and rats (30 min on tissue culture plastic dishes coated with glutaraldehyde-fixed BSA) [36] are not useful for separation of KC from pig NPC. Glass dishes coated with GA-fixed BSA were found to be the best substrate for selective adherence of KC. After KC depletion by selective adherence on this substrate, the non-adherent NPC fraction was transferred to fibronectin-coated plastic culture dishes, incubated for 15 min to allow attachment of LSEC, and washed extensively. This procedure resulted in 90% pure LSEC. Longer incubation time or too weak washing were found to yield a higher relative number of contaminating stellate cells and small PC.

Furthermore, in contrast to liver cell isolation from rat and mice, it is not feasible to remove all PC from the pig NPC suspension by neither isopycnic- or elutriation centrifugation due to the large heterogenity in both density and size of the cells. Heterogenity in density was also observed in LSEC, since the cells were found in all tested OptiPrep-layers, with densities varying from 1.038–1.086 g/ml. Moreover, LSEC were elutriated at all flow rates varying from 20–50 ml/min, indicating heterogeneity in cell size. For mass isolation of LSEC by elutriation centrifugation, we obtained purities between 80–95%, very similar to purities obtained by selective adherence.

Taken together, LSEC purified by selective adherence is faster as long as a limited number of cells are needed. Elutriation centrifugation is more time-consuming, but yields a higher cell number, with up to 1.1 × 10^9 ^purified LSEC from one pig. The addition of the detergent Pluronic acid F-68 in the elutriation buffer eliminated clotting of cells in the elutriation chamber [37], thereby allowing a higher number of cells to be loaded per cycle.

The ligands COLLA, FITC-bHA, mannan, FSA, and AGG were used to probe the endocytic activity in pig LSEC via the COLLAR, HAR, SR, mannose receptor and IgG Fc receptors, respectively. We found that all these ligands were avidly endocytosed in LSEC. Competition experiments showed that the ligands were taken up in a specific manner, via the five different categories of receptors. Moreover, morphologic pulse-chase experiments using FITC-labelled ligands to study the intracellular transport of endocytosed ligand suggested that all ligands studied, with the exception of AGG, reached lysosomal compartments within a time span of 2 h. At that time, AGG was still found in ring shaped structures which is a typical feature of early and late endosomes [33,38]. The finding that endocytosed AGG was degraded (albeit not as efficiently as other ligands), without reaching the lysosomal compartment, indicates that this ligand is processed differently than the other ligands studied. This phenomenon has also been observed by Løvdal et al. [15] who noted that rat LSEC and KC *in vitro *degraded endocytosed IgG-complexed antigen much slower than ligands for the mannose- and scavenger receptor, and that the delay was due to slow departure from early endosomes. We also observed that FITC-AGG delayed the transport of TRITC-FSA to the lysosomes when the ligands were given simultaneously. This is consistent with the observation that AGG reduces the amount of ^125^I-FSA degraded even if the total amount of endocytosed FSA was not changed. Interestingly, we observed no uptake of FITC-AGG in KC.

FITC-labelled ligands were seen concentrated in small spherical vesicles scattered over the entire cell body after a 10 min chase. These small vesicles are probably early endosomes reminiscent of small bristle coated vesicles that have been previously observed electron microscopically as vesicles with a diameter of 180 nm, located directly below the cell surface [39]. The vesicles containing FITC-bHA after a 10 min pulse at 37°C followed by a 20 min chase, are probably late endosomes (with diameter ranging between 800–1500 nm) as reported in similar studies in rat LSEC [38,39]. After a 2 h chase, the stain partly co-localized with TRITC-FSA, indicating further transport to late endosomes and lysosomes. FITC-bHA was observed in small perinuclear vesicles after a 2 h chase, and almost 30% of the endocytosed ^125^I-FITC-bHA was found as low molecular weight material, demonstrating intracellular degradation (results not shown). We stress that, although practically all cultured LSEC accumulated FITC-labelled ligands *in vitro*, not all LSEC had taken up TRITC-FSA *in vivo*. We speculate that the explanation for the heterogeneous uptake *in vivo *was due to circulatory regulation: not all sinusoids may have allowed entrance of blood at the time of injection, thus preventing LSEC from being exposed to the injected ligand in those sinusoids. This is not an unreasonable explanation, since it is known that hepatic sinusoids may regulate blood flow by a sphincter mechanism [40].

If LSEC are an important part of the reticulo-endothelial system in the body they also need a high activity of lysosomal enzymes to degrade waste material endocytosed from the circulation. Therefore, we compared the lysosomal activity in LSEC with the metabolically very active PC and the phagocytic KC. Earlier studies in rat have revealed that KC and LSEC, as compared to PC, contain higher specific lysosomal enzyme activities [41-43], and we found that all enzymes measured were present in considerably higher specific activities in LSEC than in PC. The specific activities of α-mannosidase and glucuronidase in LSEC were also higher than in KC. The high specific activities of lysosomal enzymes in LSEC are compatible with the notion that these cells are true professional scavenger cells. Moreover, studies in rat have shown that LSEC may recruit lysosomal enzymes from the circulation by endocytosis via the mannose receptor [44,45]. This may partly explain the very high specific activity of such enzymes in these cells in the pig as well.

In all our experiments we used freshly isolated cells because we believe that when cells have just been isolated from the intact organ, their *in vitro *scavenger properties resemble their *in vivo *properties. Once outside their micro-environment in the liver, rat LSEC dedifferentiate and eventually die after 2–4 days on culture dishes. During the first hours in culture only small changes in endocytic capacity of LSEC occur in so far as experiments in our laboratory have shown that pig LSEC cultivated in RPMI for 2 days retain 80% of the endocytic capacity when compared to freshly isolated cells (results not shown). Because of the short cultivation time used in our experiments, the endocytic capacity of the cells would not be influenced by mediators in the medium. But in other experiments with cultures of rat LSEC that were incubated for 18 h or more, it has been shown that inflammatory mediators like tumor necrosis factor-α and interleukin-1β enhance 2–3-fold endocytosis via the SR and mannose receptor, while COLLAR mediated endocytosis remained unaffected [46]. Also lipopolysaccharide can increase endocytosis in LSEC by stimulating the cells to release autocrine interleukin-1β. Another mediator, the nitric oxide, decreases endocytosis via the mannose receptor in rat LSEC [47]. Using interleukin-10, Knolle et al. [48] found a similar effect as that reported with nitric oxide, namely down-regulation of mannose receptor mediated endocytosis in mouse LSEC. Other potentially mediators of endocytic capacity are VEGF [49,50] and phorbol ester [51] which at least have been shown to improve maintenance of rat LSEC in culture.

## Conclusion

Our results suggest that pig LSEC are functionally very similar to rat LSEC, at least in terms of clearance activity. It is therefore highly likely that LSEC in pig (as in rat) represent the major site of elimination of an array of soluble waste molecules from the circulation. Several if not all of these waste macromolecules are harmful if allowed to accumulate in the blood. The very active endocytosis in LSEC ensures that these soluble waste macromolecules are never allowed to increase above trace levels in the circulation.

## Methods

### Chemicals and animals

1,3,4,6-tetrachloro-3α,6α-diphenylglycoluril (Iodogen), carrier-free Na^125^I, and TRITC were from Pierce, Rockford, IL, USA, Institute for Energiteknikk, Norway and ICN Biomedicals Inc., OH, USA. FITC, BSA, 4-methylumbelliferyl-substrates for fluorometric assays of lysosomal enzymes, Triton X-100, mannose, and mannan was from Sigma Chemical Co, St.Louis, MO, USA. Collagenase P was from Boehringer Mannheim, Germany. Fibronectin was kindly donated by Dr. B. Hansen, University of Tromsø, Norway. Human IgG and high molecular weight hyaluronan (Healon) were from Pharmacia, Sweden. OptiPrep was from Nycomed, Norway. Human serum albumin was from Octapharma, Ziegelbrucke, Switzerland. Monoclonal mouse anti-human desmin, clone D33, was from DAKO A/S, Denmark. Monoclonal goat anti-mouse IgG, TRITC-conjugate, was from Zymed, CA USA. Two mouse monoclonal antibodies (clones 2G6 and 2B10) against porcine macrophages were kindly provided by Dr. A. Berndt, Institute of Pathology, Friedrich Schiller University, Jena, Germany.

Castrated male piglets (*Sus scrofa domesticus*, Norwegian strain), weighing 7–8 kg, were fasted for 18 h, drinking water *ad libitum*, prior to sacrifice. Animals received care according to "Guide for the Care and Use of Laboratory Animals" prepared by the National Academy of Sciences and published by the National Institutes of Health, NIH publication 86-23 revised 1985.

### Isolation and cultivation of liver cells

The procedure for isolation of functionally intact LSEC from a single pig liver was as described [5]. Briefly, the liver was perfused with a physiological saline buffer to wash out blood cells before perfusion with a collagenase buffer to disperse the liver cells. The resulting single cell suspension was subjected to 2 × 3 min velocity centrifugation (50 *g*) to pellet PC, and the resulting supernatant was concentrated by centrifugation (850 *g*) for 10 min, mixed into a 21% OptiPrep density solution, and centrifuged for 30 min (3300 *g*). The PC and RBC were pelleted, and a layer consisting of 6 × 10^8^-3.5 × 10^9 ^non-PC were recovered. For further purification, 2.0 × 10^8 ^non-PC in HBBS solution containing 0.3 % BSA, 0.4 mM EDTA, 200 μg/ml Pluronic acid F-68, and antibiotics were introduced into a standard elutriation chamber of a JE-6B rotor (Beckman Instruments) in a J-21-type Beckman centrifuge at a flow rate of 22 ml/min and a rotor speed of 2500 rpm. The first fraction of 100 ml was enriched in stellate cells as judged by strong staining with anti-desmin antibody. The flow rate was then increased to 35 ml/min, and 150 ml was collected. This fraction contained purified LSEC characterized by their specific accumulation of FITC-labelled formaldehyde-treated serum albumin (FSA). The cells remaining in the chamber were pumped out and discharged after the centrifuge was stopped. These were mainly KC and PC. KC were identified by the immunostaining with two anti-porcine macrophage antibodies. The average numbers of LSEC grown per cm^2 ^were 2.5 × 10^5 ^in Falcon dishes (Becton Dickinson, France). An alternative separation technique was also used: the cell suspension of non-PC after the gradient centrifugation was diluted to 4 millions/ml and seeded on glass dishes at a concentration of 5 × 10^5^/cm^2 ^and allowed to attach for 15 min, at 37°C. Prior to use, the glass dishes were washed in 96% ethanol, coated with bovine serum albumin and fixed in 1% glutaraldehyde for 30 min., before being extensively rinsed in distilled water. It was mainly KC that attached well to these dishes. Poorly attached cells that were detached by gentle washing together with non-adherent cells were transferred to fibronectin-coated culture dishes, incubated for another 15 min followed by thorough washing, and supplied with fresh medium to enable attachment and spreading of viable SEC. The purity of these LSEC cultures was between 80–95%.

### Ligands

FSA was prepared by treating BSA with 10% formaldehyde in 0.2 M carbonate buffer, pH 10, for 3 days as described [52]. Aggregated gamma-globulin (AGG) was prepared by heating purified human IgG (10 mg/ml) for 30 min at 63°C [53]. Insoluble AGG was removed by centrifugation for 30 min (3300 *g*). Native triple helical collagen ((Nutacon, Leimunden, The Netherlands) was denatured to single collagen α-chains (COLLA) by incubation at 60°C for 60 min. Biotinylated hyaluronan, bHA, was prepared by incubating HA with Biotin-LC-Hydrazide (Pierce, Rockford, IL, USA) and 1-ethyl-3-[3-dimethylaminopropyl] carbodiimide hydrochloride (Sigma Chemical) in a ratio allowing a maximum of 1 out of 10 carboxyl groups per HA molecule to be labelled [54].

### Labelling of ligands with fluorochromes

FSA, AGG, collagen, bHA and mannan dissolved in sodium carbonate buffer (0.1 M, pH 9.5) were incubated with FITC or TRITC in a ligand/dye weight ratio of 5:1, at 4°C overnight. To remove unbound dye, the solutions were dialysed against PBS.

### Radiolabelling procedures

Macromolecular ligands (FSA, mannan, collagen, AGG and FITC-bHA) in PBS were labelled with Na^125^I employing Iodogen as the oxidizing agent [55]. Radiolabelled proteins and free iodine were separated by gel filtration on a PD-10 column (prepacked Sephadex G-25, Pharmacia, Uppsala, Sweden). The resulting specific radioactivity was 1–3 × 10^6 ^cpm/μg protein. To radiolabel bHA, FITC was first attached to the biotin, thereby providing an ^125^I acceptor. For separation of free iodine from radiolabelled FITC-bHA, the reaction solution was dialysed against PBS, giving a final radioactivity of 0,3 × 10^6 ^cpm/μg HA.

### Receptor specificity of ^125^I-labelled ligands

After seeding and cultivation for 2–3 h in 24 well dishes, purified cultures of LSEC were washed and supplied with fresh RPMI 1640 medium containing 1% human serum albumin and trace amounts of one of the six ^125^I-ligands (10,000–30,000 cpm per culture) and excess cold ligands. All endocytosis experiments were terminated after an incubation-period of 2 h at 37°C, by transferring the conditioned medium (200 μl), along with 500 μl PBS used for washing of the cells, to tubes containing 500 μl 20% trichloroacetic acid. Following centrifugation of the tubes, the extent of degradation was determined by measuring the radioactivity in the pellets and the supernatants, except for mannan or FITC-bHA where the trichloroacetic acid-precipitation step was omitted due to lack of degradation products being released from the cells. The ^125^I was attached to the protein core in mannan and to the FITC adduct in FITC-bHA of which both accumulate in the lysosomes, since mammalian cells do not carry degradative hydrolases for these molecules. Cell-associated radioactivity was determined by measuring the amount of ^125^I released by solubilizing washed cultures in 1% SDS. All experiments were carried out in triplicate.

### Accumulation of fluorochrome-labelled ligands

TRITC-FSA (20 mg) in 20 ml physiological saline was administered via the left external jugular vein 90 min prior to liver perfusion. Prior to use, TRITC-FSA was centrifuged at high speed and sterile-filtered in order to remove aggregates which would otherwise be taken up in KC by phagocytosis. Cultures of LSEC were established on fibronectin-coated 14 mm diameter glass coverslips for 3–4 h in serum-free growth medium. The cultures were then washed in PBS, and pulsed in fresh medium with 0.1 mg/ml FITC-ligands for 1 h at 4°C. Because FITC-labelling of proteins remove positive charges, and thus may turn proteins into negatively charged ligands for the SR [56], FITC-mannan and FITC-collagen were pulsed in the presence of 0.5 mg/ml FSA to avoid binding to SR. Endocytosis of FITC-AGG was studied in cells that had not been prelabelled with TRITC-FSA. Instead, the cells were pulsed in fresh medium with both 0.1 mg/ml FITC-AGG and TRITC-FSA for 1 h, at 4°C. After removal of unbound ligand by washing with PBS, bound ligands were chased for 10 min and 120 min in fresh pre-warmed medium at 37°C. As the only fluorochrome in the cells, FITC-bHA (0.2 mg/ml) was pulsed for 10 min at 37°C before medium change, and then chased for 20 min and 2 h. Incubations were terminated by fixation in 4% formaldehyde, and the specimens examined in a Zeiss Axioplan fluorescence microscope. Micrographs were taken with a Nikon Coolpix 4500 digital camera.

### Assay of lysosomal enzymes

Samples of PC were taken from the pellet resulting from the density centrifugation, and solubilized in 0.1 % Triton, whereas KC and LSEC samples were obtained from the solubilizates of cultures seeded on 7.5 cm^2 ^Falcon culture dishes.

The assay conditions of the six enzymes are given in Table 1. In all the enzyme assays, except for acid lipase (see below) aliquots of 100 μl substrate were incubated at 37°C with the appropriate amount of cell-solubilizates, and the appropriate length of time after which 2.0 ml of 0.5 M glycine/sodium hydroxide buffer pH 10.4 were added to stop the reaction and to develop the fluorochrome. To assay for acid lipase 10 μl of substrate was used, and 2.0 ml of 0.5 M Tris buffer pH 8.5 was added to stop the reaction.

**Table 1:** Conditions of enzyme assays.

**Enzyme**	**Conc. (mM)**	**Substrate**	**Buffer**	**pH**	**Incubation time (min)**	**Solubilisate volume (μl)**
α-Mannosidase	2.5	4-methylumbelliferyl-α-D-mannopyranoside	Phosphate/citrate (0.1 M)	4.0	120	40
Hexosaminidase	5.0	4-methylumbelliferyl-N-acetyl-β-D-glucosaminide	Phosphate/citrate (0.1 M)	4.5	30	5 10 (PC)
Glucuronidase	2.5	4-methylumbelliferyl-β-D-glucuronide	Acetate (0.1 M)	4.5	120	20 5 (LSEC)
Acid phosphatase	1.0	4-methylumbelliferyl-phosphate	Acetate (0.1 M)	4.5	30	10
Aryl sulphatase	10.0	4-methylumbelliferyl-sulfate	Acetate (0.5 M)	5.5	120	40
Acid lipase	0.3	4-methylumbelliferyl-oleate	Acetate (0.1 M)*	4.0	60	20

*Acetate (0.1 M) + 0.1% Triton X-100 + phosphatidylcholine (100 μM) + taurodeoxycholic acid (300 μM).

The fluorescence of 4-methylumbelliferone, resulting from the action of the enzymes on the various substrates, was measured in a Shimadzu RF 5000 spectrofluorometer with excitation set at 360 nm and emission at 450 nm. Enzyme activities are given in number of substrate molecules transformed min^-1^·gram protein^-1 ^under the conditions stated above.

Protein content in the samples was measured according to Lowry [57], and BSA in 0.1 % Triton was used as standard.

### Statistics

The values are expressed as: mean (standard deviation) unless otherwise noted. We used SPSS 10.0 software package (SPSS, Chicago, IL) for statistical analysis. Analysis of variance (ANOVA) was used to test whether any statistical significance existed between the three cell populations enzyme activity followed by the LSD post hoc comparison test. Probability values of p ≤ 0.05 were considered significant for all tests applied.

## Authors' contributions

KHE and GIN designed and carried out the experiments. KHE drafted the manuscript. AR and BS coordinated the study and contributed to the text of the manuscript.
